# Distinct Characteristics and Complex Evolution of PEDV Strains, North America, May 2013–February 2014

**DOI:** 10.3201/eid2010.140491

**Published:** 2014-10

**Authors:** Anastasia N. Vlasova, Douglas Marthaler, Qiuhong Wang, Marie R. Culhane, Kurt D. Rossow, Albert Rovira, James Collins, Linda J. Saif

**Affiliations:** The Ohio State University, Wooster, Ohio, USA (A.N. Vlasova, Q. Wang, L.J. Saif);; University of Minnesota Veterinary Diagnostic Laboratory, St. Paul, Minnesota, USA (D. Marthaler, M.R. Culhane, K.D. Rossow, A. Rovira, J. Collins)

**Keywords:** porcine epidemic diarrhea virus, virulent strains, S INDEL strains, complete genome analysis, phylogenetic analysis, recombination identification analysis, spike gene, PEDV variant, US strains, pigs, single nucleotide polymorphisms, open reading frame 1, ORF 1, viruses

## Abstract

Porcine epidemic diarrhea virus (PEDV), which emerged in the United States in 2013, has spread throughout North America. Limited availability of PEDV complete genomes worldwide has impeded our understanding of PEDV introduction into the United States. To determine the relationship between the North American strains and global emerging and historic PEDV strains, we sequenced and analyzed complete genomes of 74 strains from North America; the strains clustered into 2 distinct clades. Compared with the initially reported virulent US PEDV strains, 7 (9.7%) strains from 4 states contained insertions and deletions in the spike gene (S INDELs). These S INDEL strains share 99.8%–100% nt identity with each other and 96.2%–96.7% nt identity with the initial US strains. Furthermore, the S INDEL strains form a distinct cluster within North American clade II, sharing 98.6%–100% nt identity overall. In the United States, the S INDEL and original PEDV strains are co-circulating and could have been introduced simultaneously.

## Abstract 2

Sequence analysis showed heterogeneity among 74 strains and distinct molecular characteristics of highly virulent strains and variants.

Porcine epidemic diarrhea virus (PEDV) (family *Coronaviridae* family, genus *alphacoronavirus*) has an enveloped, single-stranded, positive-sense RNA genome of ≈28 kb (*1*). The 5′ two thirds of the genome contains 2 large open reading frames (ORFs), 1a and 1b, that encode 2 nonstructural polyproteins, pp1a and pp1b, that direct genome replication and transcription. The remaining PEDV genome contains ORFs specifying structural and nonstructural proteins in the following order: spike (S), ORF 3, envelope (E), membrane (M) and nucleoprotein (N) (*2*,*3*).

Porcine epidemic diarrhea (PED) was first documented in the United Kingdom in 1971 as a swine disease resembling transmissible gastroenteritis (*4*). In 1978, the etiologic agent of PED was identified in Belgium as a new coronavirus and was designated as PEDV, prototype strain CV777 (*1*). Within 2 decades, PEDV was reported in several other European countries; Hungary, Italy, Germany, France, Switzerland, and the Czech Republic (*5*). Currently, the virus causes only isolated outbreaks in Europe. In Asia, PEDV was first identified in 1982 and is now considered endemic, causing substantial economic losses to pork producers in China, South Korea, Thailand, and Vietnam (*5*). However, it was not until 2010 that massive PED outbreaks were reported in China; the outbreaks have been characterized by 80%–100% illness among infected swine herds and a 50%–90% mortality rate among infected suckling piglets (*6*–*8*).

North America was free of PEDV until its sudden and intense emergence in the United States in April 2013 (*9*). Since then, PEDV has spread rapidly across the United States, causing high rates of death among piglets and substantial economic losses (*10*–*12*). As of July 24, 2014, PEDV had been reported in 31 US states (*13*). In Canada, PEDV was first detected in January 2014 on a pig farm in Ontario Province; since then, the virus has been reported on farms in Manitoba, Prince Edward Island, and Quebec Provinces (*14*), continuing its spread throughout North America. There are no official reports of PEDV in Mexico; however, in 2013, the University of Minnesota (UM) Veterinary Diagnostic Laboratory (St. Paul, MN, USA) tested swine samples from Mexico and found them positive for PEDV.

The complete genomic sequence of the PEDV prototype strain, CV777, was determined in 2001 (*15*). A decade later, complete genomes were sequenced for several PEDV strains from China and South Korea (*6*,*8*,*15*–*22*). Comparisons of full-length genomes showed that different PEDV strains are more closely related to bat alphacoronaviruses than to other known alphacoronaviruses (*23*), suggesting that interspecies transmission of coronavirus may have occurred decades ago between bats and pigs or through an intermediate host.

 Phylogenetic analysis has shown that some PEDV strains that have reported decreased virulence in the field contain distinct insertions and deletions in the S gene (S INDELS). Soon after the emergence of PEDV in the United States, the complete genomes were determined for several strains from Colorado, Minnesota, and Iowa, USA (*10*,*24*,*25*); these PEDV strains shared ≥99.5% nt identity with strain AH2012 from China, suggesting a common ancestor for that strain and US strains (*25*,*26*). An additional 43 complete-genome PEDV sequences are now available: 16 from the United States, including a recent PEDV variant from Ohio (OH851) that contains specific S INDELs and was reported with reduced disease severity (*27*); 23 from China; and 4 from South Korea. In addition, new, complete-genome PEDV sequences are being generated and released almost monthly.

A major impediment to understanding the origin, evolution, and diversity of PEDV in the United States is the lack of complete-genome PEDV sequences worldwide. To determine the phylogenetic relationship between the new US strains and the globally emerging and historic PEDV strains, we sequenced and analyzed the complete genomes of 74 strains from North America.

## Materials and Methods

### Sample Collection and Processing

Between May 6, 2013, and February 28, 2014, porcine intestine, saliva, and feces samples and fecal swab and environment samples (N = 25,762) from North America were submitted to the UM Veterinary Diagnostic Laboratory for detection of PEDV. RNA was extracted from the samples and screened for PEDV by using previously described methods (*28*,*29*).

### Sequencing and Genome Assembly

A total of 74 PEDV-positive samples (72 from the United States, 2 from Mexico) were randomly selected for complete-genome sequencing. Sequencing and genomic assembly were conducted at the UM Genomic Center, using next-generation sequence technology as previously described (*25*). GenBank accession numbers for the PEDV strains are KJ645635–708.

### Phylogenetic Analysis

We aligned PEDV sequences by using the ClustalW (http://www.clustal.org/clustal2/) method. Phylogenetic trees for the complete genome and each gene segment were constructed by using the maximum-likelihood method with the general time reversible nucleotide substitution model and bootstrap tests of 1,000 replicates in MEGA 6.06 software (*30*). The RIP (Recombinant Identification Program; http://www.hiv.lanl.gov/content/sequence/RIP/RIP.html) was used to identify recombination points within the PEDV genome. The RIP analysis parameters were window size of 400 and confidence threshold of 90%.

## Results

### Phylogenetic Analysis of the Complete Genomes

**Figure 1:**
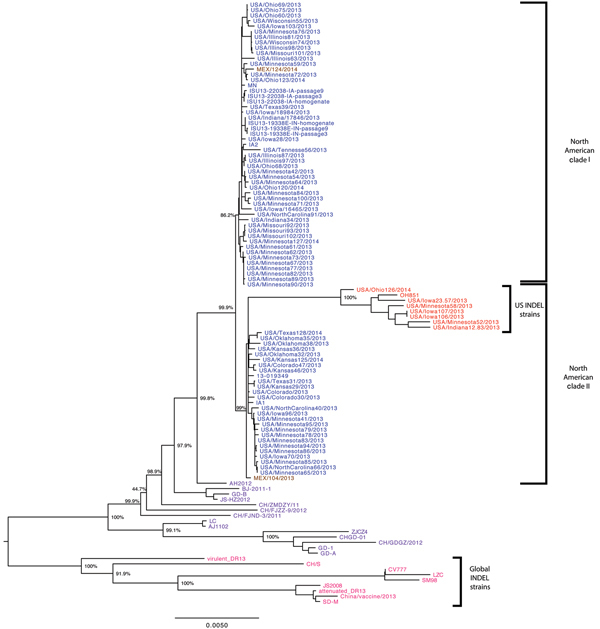
Phylogenetic tree based on complete genome sequences of 112 North American porcine epidemic diarrhea virus strains. Blue represents US non–S INDEL strains; red represents US S INDEL strains; brown represents Mexican strains; purple represents worldwide non–S INDEL strains; and pink represents global S INDEL strains. Bootstrap values are represented at key nodes. Scale bar indicates nucleotide substitutions per site. CH, China; IA, Iowa; S INDEL, insertions and deletions in the spike gene; IN, Indiana; ISU, Iowa State University; MEX, Mexico; MN, Minnesota; USA, United States of America.

A phylogenetic tree was constructed on the basis of a multiple sequence alignment of 112 sequences (the 74 PEDV sequences generated from this study and the 38 PEDV sequences available from GenBank). Most of the PEDV strains from the United States shared complete-genome nucleotide identities of 98.6%–100% and were distributed between 2 clades within tentative genogroup 2a (Figure 1) (*23*). Compared with the initially reported prototype strains from the United States, 7 (9.7%) of the 72 newly identified US strains had an S INDEL. These 7 PEDV strains were from 4 US States, shared 99.8%–100% nt identity with each other and 96.2% nt identity with the prototype North American strains, and formed a separate cluster in clade II. In addition, phylogenetic analysis of the 112 complete PEDV genomes confirmed a recent common ancestor with PEDV strains from China and all PEDV strains from North America, as was suggested previously (*25*,*26*). Because the choice and number of sequences are known to affect tree topology, we also compared the 112 PEDV complete genomes with 11 bat alphacoronavirus complete genomes as out-group sequences (data not shown) to validate the observed phylogeny of the PEDV strains from the United States. This analysis confirmed the closest recent common ancestor with strain AH2012 from China and PEDV strains from the United States, and it confirmed the presence of the 2 North American clades and the S INDEL cluster from the United States. Of the 4 states with S INDEL strains, Minnesota had the lowest detection rate for these strains (6.9%, 2 of 29 strains), followed by Ohio (14%, 1 of 7 strains); the highest rates were in Iowa (43%, 3 of 7 strains) and Indiana (50%, 1 of 2 strains).

North American PEDV clade I and clade II were uneven in size (n = 43 and n = 24, respectively) and had unequal representations from the US states; some states were represented in only 1 clade (Table). The analyzed PEDV strains from Ohio (n = 6) and Illinois (n = 4) belonged to clade I, and the PEDV strains from Oklahoma (n = 3) were grouped within clade II. PEDV strains from Minnesota constituted 42% (18 of 43 strains) of clade I and 37.5% (9 of 24 strains) of clade II, and PEDV strains from Iowa constituted 4.7% (2 of 43 strains) of clade I and 8.3% (2 of 24 strains) of clade II, corresponding with the higher sampling in these 2 states. Two isolates from Mexico, Mexico124 and Mexico104, belonged to clades I and II, respectively.

**Table:** Phylogeny and spatiotemporal distribution of porcine epidemic diarrhea virus strains detected in North America, May 2013–February 2014*

Month and year detected	Clade I	Clade II	S INDEL
June 2013			USA/Indiana12.83/2013
July 2013	USA/Iowa28/2013	USA/Kansas29/2013	
		USA/Colorado30/2013	
		USA/Texas31/2013	
		USA/Oklahoma32/2013	
August 2013	USA/Indiana34/2013	USA/Oklahoma35/2013	
September 2013	USA/Texas39/2013	USA/Kansas36/2013	
		USA/Oklahoma38/2013	
October 2013	USA/NorthCarolina40/2013	USA/Minnesota41/2013	USA/Iowa23.57/2013
	USA/Minnesota42/2013	USA/Kansas46/2013	
		USA/Colorado47/2013	
November 2013	USA/Minnesota54/2013	USA/Minnesota65/2013	USA/Minnesota58/2013
	USA/Wisconsin55/2013	USA/NorthCarolina66/2013	USA/Minnesota52/2013
	USA/Tennesse56/2013	USA/Iowa70/2013	
	USA/Minnesota59/2013	USA/Minnesota78/2013	
	USA/Ohio60/2013	USA/Minnesota79/2013	
	USA/Minnesota62/2013		
	USA/Illinois63/2013		
	USA/Minnesota64/2013		
	USA/Minnesota67/2013		
	USA/Ohio68/2013		
	USA/Ohio69/2013		
	USA/Minnesota72/2013		
	USA/Minnesota73/2013		
	USA/Wisconsin74/2013		
	USA/Ohio75/2013		
	USA/Minnesota76/2013		
	USA/Minnesota77/2013		
December 2013	USA/Illinois81/2013	USA/Minnesota83/2013	USA/Iowa106/2013
	USA/Minnesota82/2013	USA/Minnesota85/2013	USA/Iowa107/2013
	USA/Illinois87/2013	USA/Minnesota86/2013	
	USA/Minnesota89/2013	USA/Minnesota94/2013	
	USA/Minnesota90/2013	USA/Minnesota95/2013	
	USA/NorthCarolina91/2013	USA/Iowa96/2013	
	USA/Missouri92/2013	Mexico104/2013	
	USA/Missouri93/2013		
	USA/Illinois97/2013		
	USA/Illinois98/2013		
	USA/Minnesota100/2013		
	USA/Missouri101/2013		
	USA/Missouri102/2013		
	USA/Iowa103/2013		
	USA/Minnesota127/2013		
	USA/Minnesota61/2013		
	USA/Minnesota71/2013		
	USA/Minnesota84/2013		
January 2014	USA/Ohio120/2014	USA/Texas128/2014	USA/Ohio126/2014
	USA/Ohio123/2014	USA/Kansas125/2014	
	Mexico124/2014		

*S INDEL, insertions and deletions in the spike gene.

A comparison of the phylogenetic grouping and temporal distribution of the 74 PEDV strains from North America showed that clade I mostly consisted of strains collected between November and December 2013 (n = 38); only 5 clade I stains were collected during July–October 2013 (Table). In contrast, 1–6 clade II strains were collected each month throughout the year. The S INDEL strains from the United States were also detected throughout the year (1–2 strains/month). The first S INDEL strain, USA/Indiana12.83/2013, was detected in feeder pigs on June 5, 2013.

The 38 PEDV strains that were not from the United States or Mexico formed 3 distinct branches corresponding to genogroups 1a, 1b, and 2b/R, as previously reported (*26*). Other than the S INDELs, a comparison of the complete-genome sequences of all 112 PEDV strains did not show any insertions or deletions that were specific for PEDV strains from the United States; non–S INDEL mutations were represented by single-nucleotide polymorphisms (SNPs).

### Phylogenetic Analysis of ORF 1 Regions 

We also analyzed a phylogenetic tree based on the ORF 1 region of the North American and global PEDV strains (data not shown). We observed 2 identical North American clades that included, as in the complete-genome analysis, all of the strains from the United States and Mexico. The 7 S INDEL strains from the United States grouped together with strain CH/ZMDZY/11 from China but not with strain AH2012 from China, suggesting that molecular signature characteristics of the S INDEL strains are not limited to the specific S INDELS. However, the tentative parent strain, AH2012, clustered together with the non–S INDEL PEDV strains from North America.

Comparative sequence analysis of the 7 US S INDEL strains showed the presence of 99 specific SNPs in ORF 1b (10 specific to the US S INDEL strains and 89 similar to SNPs in CH/ZMDZY/11) that were not present in the other 80 North American strains. Of the 89 SNPs that were similar to SNPs in CH/ZMDZY, 13 were identical in all 7 S INDEL strains, including USA/Iowa23.57/2013 and USA/Ohio126/2014, which were slightly different from the other 5 S INDEL strains; this finding was also observed in the phylogenetic analysis (Technical Appendix Table). In addition, among 76 of the 89 specific SNPs that were similar to those in CH/ZMDZY/11, 9 were identical in 6 of the 7 S INDEL strains (excluding USA/Iowa23.57/2013 and/or USA/Ohio126/2014), 20 were identical in 5 of the 7 S INDELS strains (excluding USA/Iowa23.57/2013 and USA/Ohio126/2013), and 47 were identical in 6 of the 7 S INDEL strains (excluding USA/Ohio126/2014); however, these 76 specific SNPs were not found in any other analyzed PEDV strains from North America or in strain AH2012 from China.

### Phylogenetic Analysis of the S Genes

**Figure 2:**
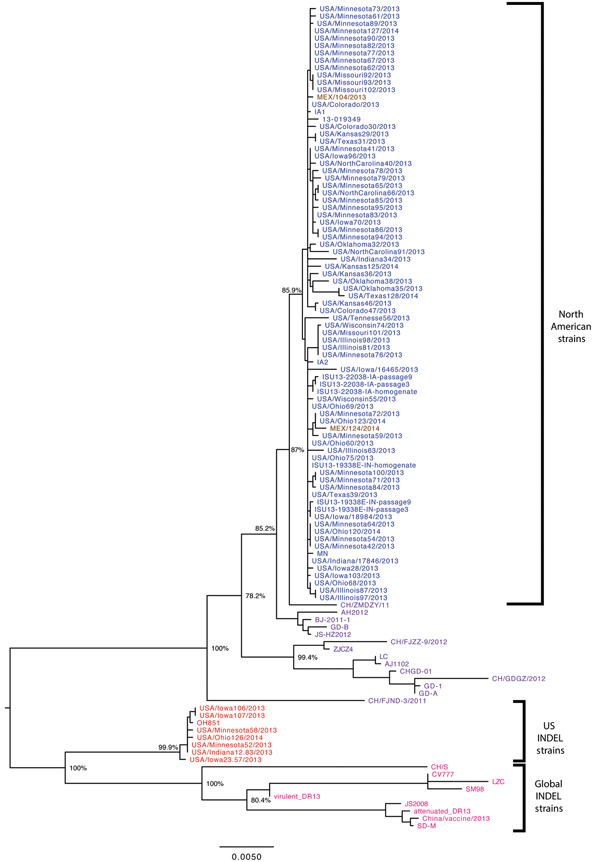
Phylogenetic tree based on the spike gene (S) sequence of 112 North American porcine epidemic diarrhea virus strains. Blue represents US non–S INDEL strains; red represents US S INDEL strains; brown represents Mexican strains; purple represents worldwide non–S INDEL strains; and pink represents global S INDEL strains. Bootstrap values are represented at key nodes. Scale bar indicates nucleotide substitutions per site. CH, China; IA, Iowa; S INDEL, insertions and deletions in the spike gene; IN, Indiana; ISU, Iowa State University; MEX, Mexico; MN, Minnesota; USA, United States of America.

We conducted S gene phylogenetic analysis on the 112 North American and global PEDV strains (Figure 2). The 7 S INDEL PEDV strains from the United States shared 99.8%–100% nt identity with each other for the entire S gene, and they shared 96.6%–97.1% nt identity with the other 67 PEDV strains from North America. In contrast to findings from the complete-genome analysis, findings from the S gene analysis indicated that strain CH/ZMZDY/11, but not strain AH2012, from China was most closely related to the non–S INDEL PEDV strains from the United States.

Phylogenetic analysis of the S gene did not show the 2 North American clades observed in the complete-genome analysis. Instead, all 112 analyzed PEDV strains were distributed between just 2 major branches, each of which had several minor sublineages. The first branch contained global PEDV sequences lacking the S INDELs; the second branch contained global PEDV S INDEL strains with US and non-US sublineages. Nucleotide sequence comparison showed that these S INDELs (1-nt, 11-nt, and 3-nt deletions at positions 167, 176, and 416, respectively, and a 6-nt insertion between positions 474 and 475) were identical in the US PEDV strains and global S INDEL strains. Moreover, 104 SNPs were identical in the S1 region (in the first 1,400 nt) of the S gene of the US and most global S INDEL strains.

The remainder of the S1 region and the full S2 region of the S gene shared high nucleotide identity among the US and global PEDV strains. Phylogenetic analysis of these regions showed that S INDEL and non–S INDEL PEDV strains from the United States were most closely related to strain CH/ZMZDY/11, but not to strain AH2012, from China (data not shown).

### Phylogenetic Analysis of ORF 3 of PEDV Strains from North America

Phylogenetic analysis of ORF 3 did not show any specific differences between the S INDEL and non–S INDEL PEDV strains from the United States (data not shown). All PEDV strains from North America grouped together, sharing 95.4%–95.7% nt identity. ORF 3 was not an immediate target region in the recent evolution of S INDEL strains in the United States. As determined by ORF 3 sequence analysis, the recent CH/ZMZDY/11 and BJ-2011-1 strains from China were most closely related to the strains from North America, and the remaining global PEDV strains formed several branches separate from the North American strains.

### Phylogenetic Analysis of E, M, and N Genes

Phylogenetic analyses of the genes for E, M, and N structural proteins showed that all PEDV strains from North America formed a monophyletic branch, sharing 99.5%–100% nt identity, and there were no major differences in these genomic regions (data not shown). The E and M gene phylogenetic analyses showed monophyletic clustering of the strains from North America with some recent strains from China: CH/FJZZ-9/2012 and CH/FJND-3/2011 for E gene and BJ-2011-1, JS-HZ2012, CH/ZMZDY/11, CH/FJZZ-9/2012, and AH2012 for M gene. In contrast, the N gene phylogenetic analysis segregated all of the North American strains from the global PEDV strains, and showed a close phylogenetic relationship between the PEDV strains from North America and some of the recent strains from China: CH/FJZZ-9/2012, CH/FJND-3/2011, BJ-2011-1, AH2012, GD-B, JS-HZ2012, and CH/ZMZDY/11.

### Potential Recombinant Origin of the S INDEL Strains

**Figure 3:**
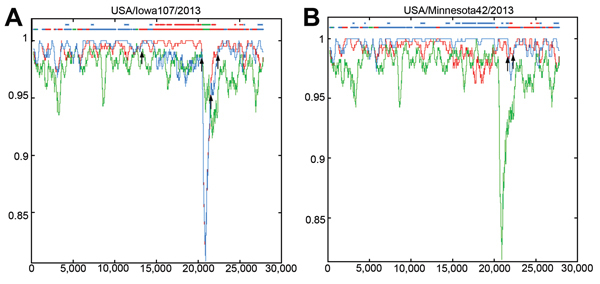
Identification of US porcine epidemic diarrhea virus (PEDV) strains with insertions and deletions in the spike gene as potential recombinant strains. At each position of the window, the query sequence USA/Iowa107/2013 (A) or USA/Minnesota42/2013 (B) was compared with background sequences for 3 strains from China (CH/ZMZDY/11, CH/s, and AH2012). The *x*-axis represents the length of the PEDV genome, and the *y*-axis represents the similarity value. The red line represents PEDV strain CH/ZMDZY/11, the green line represents CH/S, and the blue line represents AH2012. When the query sequence is similar to the background sequence(s), the homologous regions are indicated as thick dashed lines (of the corresponding color) on the top of the plot. Arrows represent potential recombination breakpoints.

To determine the involvement of recombinant events in evolution of the S INDEL strains from North America, we used RIP to compare strains from North America with the closely related ancestral strains AH2012 and CH/ZMDZY/11 from China and with the historic S INDEL strain CH/S (Figure 3). The highest overall similarity was between non–S INDEL strain USA/Minnesota42/2013 and strain AH2012; similarity was relatively consistent across most of the genome length. The lowest overall similarity was between the North American non–S INDEL strains and CH/S strain. However, sequence similarity in the S gene (excluding the hypervariable S1 region that was identical to that in AH2012) was higher between the non–S INDEL strains from the North America and CH/ZMZDY/11; this finding is consistent with the phylogenetic analysis results and indicates the presence of potential recombination breakpoints within the S gene. In contrast, the S INDEL strain USA/Iowa107/2013 appeared to possess a composite genome structure: the ORF 1a region was most similar to that of strain AH2012, the ORF 1b region was substantially more similar to that of strain CH/ZMDZY/11, the S gene S1 region was most similar to that of strain CH/S, and the S2 region was most similar to that of strain CH/ZMZDY/11. Therefore, the potential recombination breakpoints in the S INDEL strain genome may have been located between ORF 1a and ORF 1b, between S1 and S2, and between ORF 3 and E gene. The 3′ end (E, M, and, N genes) of the S INDEL and non–S INDEL strains from North America was relatively dissimilar to that of the possible parental strains (AH2012 and CH/ZMZDY/11) that were used as background sequences.

## Discussion

Despite the very recent emergence of PEDV in North America, our findings show substantial genetic heterogeneity among the strains. Our findings also confirm, as postulated by others (*25*,*26*), that strain AH2012 from China shares an ancestral strain with the non–S INDEL strains in North America. In addition, we observed an increased representation of the clade I strains at the end of 2013, which may reflect the evolution of PEDV in North America.

These findings raise several questions. First, were there multiple PEDV introductions into North America? Second, were the PEDV S INDEL variants introduced at the same time (April 2013) and are they continuing to co-circulate? Last, do the observed diversity and grouping of PEDV strains from North America represent PEDV evolution within North America, in which S INDEL strains are natural mutants of the originally detected PEDV strains in the United States? The recently identified, dating back to June 5, 2013, S INDEL PEDV strains from the United States possess several distinct molecular traits. These traits create uncertainty regarding the parental strain(s) of these PEDVs and indicate that they could have originated from multiple recombination events before their introduction into the United States. However, none of the potential parental PEDV strains for the origin of the outbreaks in North America has been identified worldwide. Overall, the findings from our phylogenetic and recombination identification analyses did not support the hypothesis of a new PEDV introduction into the United States after April, 2013; instead, our findings highlighted the possibility of multiple parental PEDV strains introduced into North America at the same or similar time.

Identical INDELs and SNPs in the highly immunogenic S1 region of the S gene of the global and the US S INDEL PEDV strains may have been generated through a long chain of recombination events or similar evolutionary mechanisms that enabled PEDV to evade the host immune response. Similar evolutionary mechanisms could have resulted in de novo generation of these specific INDELs in each country-specific PEDV pool. In addition, these S INDELs and the associated SNPs in the hypervariable S1 region of the S gene may represent a stable molecular pattern possibly associated with a less virulent phenotype. The reportedly less virulent variant US PEDV strain, OH851, contained these S INDELs and the SNPs in the ORF 1b region (*27*). These S INDELs are reportedly associated with decreased virulence and are relatively infrequent (in ≈9.5%–14% of PEDV strains), suggesting that they may also be associated with decreased transmission rates, compared with rates for typical highly virulent PEDV strains. The immunologic cross-protection in pigs between S INDEL and non–S INDEL PEDV strains in the United States is unknown, but in Asia, the historic S INDEL vaccines (CV777 lineage) failed to protect against the virulent PEDV strains that emerged after 2010 (*5*). Therefore, whether S INDEL PEDV generation represents a mechanism of adaptation to a partially immune pig population after initial infection with highly virulent non–S INDEL strains is unclear. Most historic (1978–2008) PEDV strains from Europe and Asia contain these INDELs/SNPs in their S protein, suggesting that these changes could contribute to their long-time circulation (endemic) in swine populations, potentially causing only mild disease.

The S INDELs and specific SNPs in the PEDV strains from North America appeared in strong association with a distinct pattern of specific SNPs in the ORF 1b region, which shares a common recent ancestor with strain CH/ZMZDY/11, but the S1 region of the S gene could have been provided by another ancestral strain. A comparison of the complete genome sequences of strain DR13, a virulent PEDV strain from Korea, and its attenuated counterpart showed specific changes in ORFs 1ab and 3 and in S and E genes of the attenuated strain; as a result of these changes, the ORFs and genes in the attenuated strain are shorter than those in the virulent strain (*20*). However, in contrast to the findings for the virulent–attenuated DR13 strain pair and the strong evidence of the role of ORF 3 alterations in the pathogenesis of or cell culture adaptation of feline infectious peritonitis virus (*31*), we did not observe any of the following: characteristic changes in the ORF 3–E gene region, other than S INDELs; a potentially recombinant ORF 1b region; or any reports of decreased severity of PEDV in the field. The higher level of nucleotide conservation of the North American PEDV strains in the E and M genes and greater genetic variability of N gene may be representative of geographic specificity rather than virulence. The mechanisms governing the virulence of PEDV (or coronaviruses in general) may be redundant, or the S INDEL strains may be an intermediate between those of virulent and attenuated PEDVs (e.g., lower virulence).

Our findings provide evidence of the complex and rapid evolution of PEDV strains in North American swine herds. In addition, our findings suggest that the S INDEL strains, which are likely of recombinant origin, may represent complementary PEDV mechanisms that enable the spread and persistence of the virus in swine populations after exposure to highly virulent PEDV strains. This possibility should be confirmed by thoroughly assessing the immune responses to PEDV in the US swine that were sources of the S INDEL PEDV variant strains and by conducting experimental pig inoculation and cross-protection studies. Active epidemiologic surveillance and immunologic studies are urgently needed to understand PEDV evolution worldwide and to develop optimal PEDV vaccines.

Technical Appendix Single-nucleotide polymorphisms (n = 89) that are identical in open reading frame 1b of porcine epidemic diarrhea virus strain CH/ZMDZY/11 from China and the 7 porcine epidemic diarrhea virus stains from the United States that have insertions and deletions in the spike gene.
