# Supplementary material for: Distinct Characteristics and Complex Evolution of PEDV Strains, North America, May 2013–February 2014
Source: Emerg Infect Dis. 2014 Oct;20(10):1620–8. doi: 10.3201/eid2010.140491 (PMC4193278; doi:10.3201/eid2010.140491)
Supplement: Technical Appendix — Single-nucleotide polymorphisms (n = 89) that are identical in open reading frame 1b of porcine epidemic diarrhea virus strain CH/ZMDZY/11 from China and the 7 porcine epidemic diarrhea virus stains from the United States that have insertions and deletions in the spike gene. [file 14-0491-Techapp-s1.pdf]

Article DOI: http://dx.doi.org/10.3201/eid2010.140491

# Distinct Characteristics and Complex Evolution of PEDV Strains, North America, May 2013–February 2014

## Technical Appendix

Technical Appendix Table. SNPs in open reading frame 1b that were identical in 7 S INDEL strains from the United States and in strain CH/ZMDZY/11 from China but not present in non–S INDEL strains from North America or in strain AH2012 from China*

| Nucleotide position | SNP |
|---|---|
| Present in 6/7 US S INDEL strains (excludes USA/Iowa23.57/2013 (n = 9) | |
| 14879 | C |
| 15062 | C |
| 15068 | A |
| 15116 | C |
| 15251 | C |
| 15257 | T |
| 15264 | T |
| 15266 | G |
| 15374 | G |
| Present in 5/7 US S INDEL strains (excludes USA/Iowa23.57/2013 and USA/Ohio126/2014) (n = 20) | |
| 15509 | T |
| 15629 | T |
| 15689 | G |
| 15758 | G |
| 15773 | A |
| 15824 | C |
| 15902 | T |
| 16191 | C |
| 16220 | C |
| 16391 | T |
| 16412 | C |
| 16454 | C |
| 16508 | T |
| 16730 | C |
| 16829 | C |
| 16889 | C |
| 16962 | T |
| 17084 | T |
| 17141 | T |
| 17345 | C |
| Present in 6/7 US S INDEL strains (excludes USA/Ohio126/2014) (n = 47) | |
| 17378 | C |
| 17429 | G |
| 17606 | T |
| 17622 | C |
| 17651 | G |
| 17654 | T |
| 17657 | T |
| 17660 | G |
| 17672 | A |
| 17693 | T |
| 17759 | C |
| 17780 | C |

| Nucleotide position | SNP |
|---|---|
| 17870 | C |
| 17876 | C |
| 17879 | T |
| 18005 | A |
| 18143 | A |
| 18167 | C |
| 18209 | C |
| 18272 | C |
| 18332 | T |
| 18353 | C |
| 18458 | C |
| 18485 | C |
| 18521 | T |
| 18530 | C |
| 18551 | T |
| 18578 | A |
| 18608 | C |
| 18635 | C |
| 18656 | G |
| 18668 | C |
| 18686 | C |
| 18713 | C |
| 18773 | T |
| 18782 | G |
| 18848 | C |
| 18950 | T |
| 18956 | C |
| 19022 | C |
| 19052 | T |
| 19055 | C |
| 19100 | T |
| 19226 | C |
| 19260 | T |
| 19316 | T |
| 19376 | C |
| Present in 7/7 US S INDEL strains (n = 13) | |
| 19418 | T |
| 19439 | A |
| 19484 | T |
| 19574 | C |
| 19592 | T |
| 19622 | G |
| 19632 | A |
| 19634 | T |
| 19817 | T |
| 19874 | T |
| 20069 | G |
| 20108 | C |
| 20207 | T |

*S INDEL, insertions and deletions in the spike gene; SNPs, single-nucleotide polymorphisms.
